# EPHA2 Interacts with DNA-PK_cs_ in Cell Nucleus and Controls Ionizing Radiation Responses in Non-Small Cell Lung Cancer Cells

**DOI:** 10.3390/cancers13051010

**Published:** 2021-02-28

**Authors:** Vitaliy O. Kaminskyy, Petra Hååg, Metka Novak, Ákos Végvári, Vasiliki Arapi, Rolf Lewensohn, Kristina Viktorsson

**Affiliations:** 1 Department of Oncology/Pathology, Karolinska Institutet, Stockholm, Sweden; petra.haag@ki.se (P.H.); metka.novak@nib.si (M.N.); valia_arapi@yahoo.gr (V.A.); rolf.lewensohn@ki.se (R.L.); 2 Department of Genetic Toxicology and Cancer Biology, National Institute of Biology, 1000 Ljubljana, Slovenia; 3 Division of Physiological Chemistry I, Department of Medical Biochemistry & Biophysics, Karolinska Institutet, SE-171 77 Stockholm, Sweden; akos.vegvari@ki.se; 4 Theme Cancer, Patient Area Head and Neck, Lung, and Skin, Karolinska University Hospital, SE-171 64 Solna, Sweden

**Keywords:** non-small cell lung cancer, EphA2, DNA damage response, DNA-PK**_cs_**, ionizing radiation

## Abstract

**Simple Summary:**

Despite the introduction of targeted therapies against genomic drivers in non-small cell lung cancer (NSCLC), e.g., mutated epidermal growth factor receptor (EGFR) and EML4-ALK fusion or immune checkpoint blockade, many patients are receiving radiation therapy (RT). Thus, RT is important for the treatment of advanced NSCLC, where it is used alone or combined with targeted agents or chemotherapy. Unfortunately, a large fraction of the NSCLC cases show resistance to ionizing radiation (IR), calling for further understanding of the underlying mechanisms and novel ways to IR sensitization. Here, we demonstrate a novel function of Ephrin type-A receptor 2 (EphA2) on the DNA damage response (DDR) pathway that controls cellular IR response. Moreover, we show that EphA2 can be targeted for IR sensitization of resistant NSCLC cells. Thus, we suggest that further understanding of this mechanism may allow for new RT sensitization approaches to treat LC.

**Abstract:**

Ephrin (EFN)/Erythropoietin-producing human hepatocellular receptors (Eph) signaling has earlier been reported to regulate non-small cell lung cancer (NSCLC) cell survival and cell death as well as invasion and migration. Here, the role of Ephrin type-A receptor 2 (EphA2) on the DNA damage response (DDR) signaling and ionizing radiation (IR) cellular effect was studied in NSCLC cells. Silencing of EphA2 resulted in IR sensitization, with increased activation of caspase-3, PARP-1 cleavage and reduced clonogenic survival. Profiling of EphA2 expression in a NSCLC cell line panel showed a correlation to an IR refractory phenotype. EphA2 was found to be transiently and rapidly phosphorylated at Ser897 in response to IR, which was paralleled with the activation of ribosomal protein S6 kinase (RSK). Using cell fractionation, a transient increase in both total and pSer897 EphA2 in the nuclear fraction in response to IR was revealed. By immunoprecipitation and LC-MS/MS analysis of EphA2 complexes, nuclear localized EphA2 was found in a complex with DNA-PK_cs_. Such complex formation rapidly increased after IR but returned back to basal level within an hour. Targeting EphA2 with siRNA or by treatment with EFNA1 ligand partly reduced phosphorylation of DNA-PK_cs_ at S2056 at early time points after IR. Thus, we report that EphA2 interacts with DNA-PK_cs_ in the cell nucleus suggesting a novel mechanism involving the EphA2 receptor in DDR signaling and IR responsiveness.

## 1. Introduction

Genomic analyses of non-small cell lung cancer (NSCLC) have revealed precision medicine targets, e.g., mutated epidermal growth factor receptor (*EGFR*) and *ALK* fusions [1,2,3]. Yet a large fraction of metastatic- or advanced NSCLC cases are receiving radiation therapy (RT) either alone or combined with chemotherapy. RT is also an important treatment modality for tumor lesions that progress upon EGFR or ALK Tyrosine kinase inhibitors (TKIs) or immune checkpoint inhibitors (ICI) (reviewed in [4,5]). Hence further exploration of ionizing radiation (IR) responses in NSCLC cells are warranted to identify novel ways for RT sensitization. IR-induced DNA double strand breaks (DNA DSBs) triggers activation of the DNA damage response (DDR) signaling network [6,7]. Two complementary pathways, homologous recombination (HR) and non-homologous end joining (NHEJ), are important for handling DNA DSBs repair [7]. In both HR/NHEJ, DNA DSBs are recognized by protein complexes, i.e., the MRN complex or the DNA-PK subunits Ku70/Ku80, respectively [8]. Subsequently the ataxia–telangiectasia mutated (ATM) and DNA-dependent protein kinase, catalytic subunit (DNA-PK_cs_) kinases associate, get autophosphorylated on multiple residues, e.g., ATM at S1981, DNA-PK_cs_ at S2056 and cause phosphorylation of H2A histone family member X (H2AX) on S139 into γH2AX [6,7,9]. The chromatin-attached γH2AX then serves as a scaffold for DDR signaling components allowing for DNA DSBs repair to take place [10].

Plasma membrane associated receptor tyrosine kinases (RTKs) are oncogenic drivers [7,11] but may also influence cellular transcription and DNA repair [7,11]. Thus, IR has been reported to cause autophosphorylation of both EGFR and insulin growth factor receptor 1(IGF-1R), thereby activating pro-survival kinases, e.g., mitogen activated protein kinase (MAPK) and phosphoinositide-3-kinase (PI3K)/Akt, which can impair IR-induced cell death signaling [7,11,12]. Moreover, EGFR and IGF-1R may also directly influence cellular DNA DSBs’ repair capacity by interacting with the NHEJ component DNA-PK_cs_ [7,11,12,13,14,15,16,17,18]. Accumulation of unresolved DNA damage can subsequently lead to activation of different cell death routes [19].

The erythropoietin-producing human hepatocellular (Eph) receptor family and their ligands Ephrins (EFNs) control multiple tumor characteristics including proliferation, cell death, induction of angiogenesis, invasion and metastatic spread [20,21,22,23]. Binding of the cell adherent EFNA/B ligands to Eph A/B receptors results in forward Eph RTK tyrosine phosphorylation, alteration in activation of pro-survival kinases, e.g., PI3K/Akt, but also reversed signaling through the EFN A/B ligands [20,21,22,23]. Multiple EphAs, e.g., EphA2, EphA4, EphA5, have been shown to regulate various cancer hallmarks of NSCLC [24,25,26,27,28,29,30,31,32,33,34,35,36]. Thus Brannan et al. demonstrated that inhibition of EphA2 expression blocked NSCLC cell colony formation as well as migration *in vitro*, but also that high EphA2 expression in NSCLC specimen was linked to both recurrence and metastasis formation [26]. A similar effect of EphA2 on proliferation was also reported by us [32] as well as by Amato et al. which furthermore demonstrated that targeting EphA2 in *KRAS* or *EGFR* mutant NSCLC impaired tumor growth in mice and facilitated apoptotic response by decreasing S6K1-mediated phosphorylation of BAD [27]. The role of EphA2 in tumors is complex as it may be either tumor suppressive or tumor promoting depending on the binding of different EFN ligands which influence both classical tyrosine dependent signaling as well as non-canonical activation as illustrated in multiple tumor types, e.g., glioblastoma, breast cancer, malignant melanoma, prostate cancer and ovarian carcinoma [20,21,22,23,37,38,39,40,41,42,43]. Phosphorylation of EphA2 on S897 has also been reported in NSCLC cells by us and others [27,30,32,44]. In particular, it was shown that phosphorylated Ribosomal protein S6 kinase (RSK) and pEphA2 S897 co-localize in NSCLC tumor specimen and was linked to poor patient prognosis [30]. Moreover, pEphA2 S897 was nicely demonstrated to regulate EGFR-targeted therapy refractoriness by influencing proliferation and cell death signaling [28,31,45,46]. Adding further to the multifaceted role of EphA2 in NSCLC, it was reported that EphA2 associates with PLCγ1 into a signaling circuit that influenced tumor cell growth [33]. Interestingly, it was also recently revealed that VEGFR2-targeted therapy influenced a novel VEGFR2 and EphA2 association in NSCLC cells with a downstream effect on RSK as well as on pEphA2 S897 signaling, invasion and metastatic potential [34].

We earlier identified the EFNB3 ligand as a driver of IR refractoriness in NSCLC cells and silencing of EFNB3 expression sensitized cells to IR-induced cell death [47]. Several Ephs have indeed been explored for their role in cellular IR response, e.g., EphA2 in hepatic cellular cancer [48], EphA4 in colorectal cancer [49], EphB2 in medulloblastoma [50] and EphB4 in head and neck squamous cell carcinoma [51]. Interestingly, EphA5 has been demonstrated to interact with the DDR protein ATM and in this way influence IR-induced DNA damage signaling in NSCLC cells [36]. Moreover, an increased level of pEphA2 S897 was also evident in NSCLC cells upon irradiation, and it was revealed that both RSK and MEK contributed to such a signal [52].

Given the prominent role of EphA2 signaling in NSCLC [24,25,26,27,28,29,30,31,32,33,34] and the recent reports on EphA2 phosphorylation on S897 after IR [52], we here analyzed whether EphA2 plays a role in DDR signaling of NSCLC cells. In our study we showed that EphA2 is localized to the cell nucleus and that by reducing EphA2 expression NSCLC can be sensitized to IR-induced cell death. By immunoprecipitation of EphA2 complexes and LC-MS/MS analysis, we identified DNA-PK_cs_ as a novel interacting partner of EphA2. Our data suggest a novel mechanism involving EphA2 in cellular DDR signaling with an effect on IR responsiveness. Thus, our study supports EphA2-targeting as a RT sensitizing strategy of tumors.

## 2. Materials and Methods

### 2.1. Cell Lines and Cell Culture

A panel of NSCLC cell lines, adenocarcinomas (H23, H1299, H460, A549, *EGFR*-mutant HCC827, H1975 (American Type Culture Collection (ATCC, Manassas, VA, USA) or PC-9 (Sigma-Aldrich Sweden AB, Stockholm, Sweden) and large cells (U-1810, kind gift from Uppsala University [53]) were used. For cell culture, RPMI-1640 medium supplemented with 10% heat-inactivated fetal bovine serum (FBS) and 2 mM L-glutamine (Gibco, Life Technologies, Stockholm, Sweden) was used. The IR sensitivity of the cell lines has previously been reported as survival fraction 2 Gy (SF2) in clonogenic assays, and these results were used in the current work. SF2 values: H23-0.17, A549-0.71, H460–0.53, H1299–0.97, HCC827–0.35, H1975–0.47, PC-9–0.42 and U-1810–0.88 [54,55,56].

### 2.2. Ionizing Radiation Exposure, Drug-or Biological Agent Treatments

Irradiation (IR) was carried out on adherent cells with indicated doses using a Precision X-Ray inc source (6.85 Gy/min, X-RAD 225 XL, North Branford, CT, USA). The non-irradiated cells were handled in parallel to the irradiated cells; thus, they were seeded at the same density, incubated for the same time at room temperature or in the cell incubator to avoid any bias in DDR signaling, etc. In some experiments, pretreatment with the DNA-PK_cs_ inhibitor NU7026 (#N1537, Sigma-Aldrich, 10 µM, 1 h) was applied. In the studies of EphA2 phosphorylation, cells were treated with TNFα (#H8916, Sigma-Aldrich, 20 ng/mL) or rhEFNA1 (#6417-A1, R&D systems, Biotechne, Abingdon, United Kingdom 100 ng/mL) alone or pretreated for either 10 min or 2 h before IR.

### 2.3. Immunoblotting

For preparation of total cell extracts, cells were lysed in RIPA buffer (50 mM Tris-HCl pH 7.4, 150 mM NaCl, 1% Triton-X100, 1 mM EDTA, 0.1% SDS) supplemented with protease/phosphatase inhibitors (complete Mini; Phos STOP, Sigma-Aldrich), incubated on ice for 15 min and cell debris was cleared by centrifugation (13,000 rpm, 15 min). The protein concentration was measured in the resulting supernatants by BCA assay (Pierce™ BCA Protein Assay, Thermo Fisher Scientific, Stockholm, Sweden). For protein profiling, 40–50 µg of total protein was separated on 4–12% Bis-Tris or 3–8% Tris-Acetate NuPAGE^®^ gels (Thermo Fisher Scientific). NuPAGE^®^ MES SDS running buffer (#NP0002) and NuPAGE^®^ TRIS-Acetate SDS running buffer (#LA0041) (both from Thermo Fisher Scientific) were used. Proteins were blotted onto Nitrocellulose Membrane (LI-COR GmbH, Bad Homburg, Germany) in 10% methanol-containing transfer buffer. Odyssey^®^ blocking buffer (LI-COR GmbH)/TBST 1:1 was used for blocking membranes, which subsequently were probed with primary antibodies overnight (at +4 °C), rinsed in TBS-T followed by donkey anti-mouse or goat anti-rabbit secondary antibodies conjugated with IRDye 600/800 (#926-68072; #926-32211; LI-COR GmbH). Resulting signals from secondary Abs were monitored and quantified on the Odyssey^®^ Sa Infrared Imaging System (LI-COR GmbH). The primary antibodies used were cleaved caspase-9 (ASP330) (#9501S), cleaved caspase-3 (ASP175) (#9661), pEphA2 S897 (#6347), total EphA2 (#6997), pATM S1981 (#4526), pEGFR Y1068 (#3777), pRSK S380 (#11989), total EGFR (#4267), pH2AX S139 (#9718) (all from Cell Signaling Technology, BioNordica AB, Stockholm, Sweden), pDNA-PK_cs_S2056(#ab18192), pDNA-PK_cs_S2056 (#ab124918), total ATM (#ab32420) (all from Abcam, Cambridge, United Kingdom); pEphA2 Y588 (#LS-C178069, LSBio, WA, USA); and PARP-1 (#sc-7150) (Santa Cruz Biotechnology, Santa Cruz, CA, USA); EphA2 (#37-4400), pKAP-1 Ser824 (#A300-767A-M), pRPA32 (#A300-245A-M) and total DNA-PK_cs_ (#Ab-4 cocktail) (all ordered from Thermo Fisher Scientific). Differences in loading were controlled by antibodies against β-actin (#ab8227, Abcam) or β-tubulin (#T7816, Sigma Aldrich AB, Stockholm, Sweden).

### 2.4. Subcellular Fractionation

To assess localization of EphA2, RSK, EGFR and DDR signaling components prior and post IR, cytosolic and nuclear fractions of H1975, U-1810 or A549 cells were obtained using the NE-PER Nuclear and Cytoplasmic Extraction Reagents Kit (#78833,Thermo Fisher Scientific) per manufacturers’ instructions. CER-I buffer supplemented with protease/phosphatase inhibitors (complete Mini; Phos STOP, Sigma Aldrich) was added to cell pellets which were incubated on ice for 10 min. Subsequently CER-II buffer was added for 1 min on ice followed by centrifugation (14,000 rpm, 10 min) generating cytosolic fractions. The cell pellets were rinsed in ice-cold PBS (3 times) and re-suspended in NER buffer (with protease/phosphatase inhibitors as above). After a 40 min incubation on ice, samples were cleared from non-solubilized material by centrifugation (14,000 rpm, 10 min) with nuclear protein extracts harvested as supernatants. The cytoplasmic-and nuclear fractions were studied by immunoblotting in which purity and equal loading of each fraction was confirmed by detecting β-tubulin (#T7816,Sigma-Aldrich) and cAMP response element-binding protein (CREB) (#9197, Cell Signaling Technology) for cytosolic and nuclear fractions, respectively.

### 2.5. Immunoprecipitation

Immunoprecipitation (IP) was carried out on total or nuclear/cytosolic fractions of NSCLC cell lysates prior to and post IR treatment as described in Section 2.4. The IP was performed using Dynabeads Protein G Immunoprecipitation Kit (#10007D, Life Technologies, Carlsbad, CA, USA). For each IP, 2 µg antibody/sample was conjugated to 50 µL of magnetic beads for 30 min at RT. Total cell lysates (approx. 1 mg of total protein), nuclear extracts (up to 300 µg of protein per IP) or cytosolic extract (approx. 700 µg protein per IP) were incubated with antibody-bead complexes for 6 h or overnight at +4 °C. For each IP experiment, the BCA Protein Assay Kit (#23227, Fisher Scientific) were used to reveal protein concentration and concentration equalized among the samples, and then the same volumes of protein extracts were used for IPs. For IP, the following antibodies were used: EphA2 (#6997, Cell Signaling Technology) and DNA-PK_cs_ (#Ab-4 cocktail, Thermo Fisher Scientific). To control for potential unspecific binding, half of the volume of the untreated cell lysate was incubated with 2 µg isotype normal mouse IgG (#12-371, Millipore, Temecula, CA, USA) or rabbit (DA1E) IgG (#3900, Cell Signaling Technology) conjugated to 50 µL of magnetic beads and the other half of the lysate was used for IPs with either DNA-PK_cs_ or EphA2 antibodies. Captured complexes were eluted with 20 µL elution buffer provided in the kit. All immune complexes were analyzed by immunoblotting by adding NUPAGE LDS Sample Buffer and Sampling Reducing Agent and incubating for 10 min at 70 °C with further process as described in Section 2.3. A subset of the IP samples were also studied by mass spectrometry as indicated below.

### 2.6. siRNA Transfection

The effect of EphA2 siRNA knockdown alone or combined with IR was studied in NSCLC U-1810, H1975 and A459 cells. Cells were seeded (0.5 × 10^6^ cells/60-mm dish) and the next day transfected with either an ON-TARGETplus non targeting pool (D-001810-10), ON-TARGETplus smart pool siRNAs against EphA2 (5′-UGAAUGACAUGCCGAUCUA-3′, 5′-GAAGUUCACUACCGAGAUC-3′, 5′-CAAGUUCGUUGACAUCGUC-3′, 5′-UCACACACCCGUAUGGCAA-3′ (Thermo Fisher Scientific Inc., Hanover Park, IL, USA), single oligo siRNA targeting EphA2 (5′-GGUGCACGAAUUCCAGACGTT-3′) or negative control siRNA (#4457287) (both form Life Technologies, Stockholm, Sweden). siRNAs were diluted in Opti-MEM medium (Gibco, Life Technologies, Stockholm, Sweden) and then mixed with Dharmafect 1 reagent (Dharmacon, Thermo Fisher Scientific) per manufacturers´ instructions. The final concentration of siRNA used was 25 nM.

### 2.7. Clonogenic Survival Assay

The colony formation ability of U-1810 or H1975 cells after treatment with siRNA against EphA2 or scrambled siRNA was examined alone or combined with IR. For that purpose, cells were transfected with either siRNA (see Section 2.6) and irradiated with 4 Gy at 24 h post transfection. One hour after IR, cells were trypsinized, and 200 cells/well were seeded in 6-well plates and allowed to divide and form colonies during a 10-day (U-1810) or 14-day (H1975) period. Colonies were visualized by crystal violet staining, and the total number of colonies per well was counted. Clonogenic survival is presented as a percentage of colonies that are formed in treated samples in relation to non-irradiated samples transfected with scramble siRNA. Each transfected sample was seeded for colony formation in triplicates.

### 2.8. Immunofluorescence Analyses

Cells were seeded in chamber slides (#354108, FALCON, Staten Island, NY, USA) at total of 30,000 cells/well and were the following day irradiated. At 1 h post IR, slides were fixed in 4% formaldehyde solution in PBS (10 min, RT) followed by rinsing in PBS (3x). Cells were permeabilized after fixation for 10 min with 0.25% of Triton-X100 in PBS and blocked for 1 h in 2% BSA-containing PBS. The following primary antibodies were diluted in 2% BSA-containing PBS and the slides were incubated overnight at +4 °C: DNA PK_cs_ (#Ab-4 cocktail, Thermo Fisher Scientific), EphA2 (#S6997) and pEphA2 S897 (#6347) (both from Cell Signaling Technology). After rinsing in PBS, secondary goat anti-mouse (1:500; #A11005) or goat anti-rabbit (1:500; # A11034) (both from Thermo Fisher Scientific) antibodies were added for 2 h at room temperature. Slides were mounted in VECTASHIELD Mounting Medium with DAPI (Vector Laboratories, BioNordica Sweden AB) and staining was evaluated using an Axioplan 2 (Zeiss) fluorescent microscope, with images captured at 20×–100× magnification using a CCD camera (Hamamatsu, Japan).

### 2.9. Mass Spectrometric Analysis of Immunoconjugates

For the LC-MS/MS assessment, immunoconjugates were directly digested by trypsin while complexes were remaining on the magnetic beads. The resulting peptides were purified and resuspended in 2% acetonitrile (ACN), 0.1% formic acid (FA) in a total volume of 15 µL. Nano-flow LC-MS/MS was performed on an Ultimate 3000 UHPLC chromatographic system (Thermo Fisher Scientific) coupled on-line to a Q Exactive™ HF hybrid Quadrupole-Orbitrap™ mass spectrometer (Thermo Fisher Scientific) by injecting 2 µL of the sample. The tryptic peptides were separated on a 50 cm long heated (55 °C) C-18 Easy-Spray™ column (Thermo Fisher Scientific) using an organic gradient 4–26% B in 120 min at 300 nL/min flow rate (solvent A: 2% ACN, 0.1% FA; solvent B: 98% ACN, 0.1% FA). Survey mass spectra were acquired at mass resolution of 120,000 in the range of *m*/*z* 350–1600. MS/MS data of the 17 most intensive precursors were obtained at a resolution of 30,000 by higher-energy collisional dissociation (HCD) with 28% normalized collision energy. An in-house written Raw2mgf program was used to convert the MS raw files into Mascot Generic Format (mgf). For protein identification the mgf files were searched against the SwissProt database (HUMAN) with Mascot Server search engine (v2.5.1, MatrixScience Ltd., London, UK) allowing up to two missed cleavage sites for trypsin, a mass tolerance of 10 ppm and 0.02 Da for the precursors and HCD fragment ions, respectively. Carbamidomethylation of cysteine was used as fixed modification, while asparagine and glutamine deamidations and methionine oxidation were dynamic.

### 2.10. Statistical Analysis

The association between IR sensitivity of the NSCLC cell lines given as published SF2 values and total EphA2 expression was analyzed by GraphPad Prism 8 (GraphPad Software, Inc., LA Jolla, San Diego, CA, USA). The in-built software tool was used to reveal Pearson’s correlation coefficient. GraphPad software was also applied to plot clonogenic data with mean ± SD and results from three technical replicative plates shown.

## 3. Results

### 3.1. Expression of EphA2 in NSCLC Cells Is Negatively Associated with Response to IR

**Figure 1:**
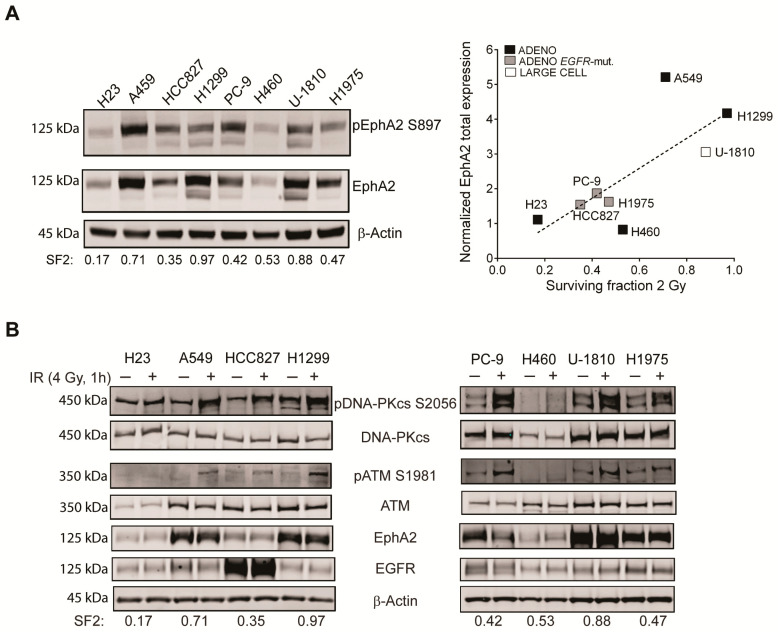
Expression and phosphorylation of EphA2 at S897 in NSCLC cells in relation to IR sensitivity and IR response. (**A**) The level of pEphA2 S897 or total EphA2 were studied by immunoblotting in a NSCLC cell line panel with β-Actin used as a loading control (blot shown corresponds to EphA2 total blot). Published SF2- values for the different cell lines are shown. Total EphA2 was quantified and expression given after correction for loading differences. This data was analyzed in relation to SF2 values. (**B)** NSCLC cell lines were profiled at baseline or 1 h post IR (4 Gy) for phosphorylation of DNA-PK_cs_ at S2056, ATM at S1981, or expression of total DNA-PK_cs_, ATM, EphA2 and EGFR. To control for loading differences β-Actin was used.

EphA2 is an important signaling component in NSCLC, which regulates proliferation, cell death, migration, invasion and metastasis [24,25,26,27,28,29,30,31,32,33,34]. There is also evidence that EphAs/EphBs influence IR response in tumor cells of different origin [36,48,49,50,51,52]. In particular, it was shown that IR triggers a transient increase in phosphorylation of EphA2 at S897 in NSCLC cells [52]. Such activation of EphA2 has previously been seen in NSCLC cells and been linked to pro-survival and metastatic potential as well as to targeted therapy response [27,28,30,32,34]. Given these findings, we here tested a hypothesis that EphA2 could be involved in regulating DDR pathways. First the relationship between EphA2 expression and IR-sensitivity was analyzed (Figure 1). For this purpose, a NSCLC cell line panel with different IR sensitivity (reported as Surviving Fraction 2 Gy (SF2) [54,55,56]) was profiled for phosphorylation of EphA2 at Ser 897 or expression of total EphA2 (Figure 1A). Three of the cell lines (A549, H1299 and U-1810) with the lowest IR response (SF2 above 0.7) were found to have the highest total EphA2 expression, while the most IR sensitive one H23 (SF2 of 0.17) displayed the lowest EphA2 level. The *EGFR*-mutant cell lines (HCC827, PC-9 and H1975) expressed relatively moderate levels of EphA2 and were previously reported to have an SF2 between 0.4 and 0.6, illustrating a partial IR responsive phenotype. Analyses of total EphA2 as related to SF2 values revealed a Pearson coefficient value of 0.74, indicating a strong correlation between a high EphA2 expression and resistance to IR (Figure 1A, right panel). Similar to the expression of total EphA2, phosphorylation of EphA2 at S897 was high in the NSCLC cell lines with the highest SF2 values (A549, H1299, U-1810) but also evident in some of the more IR-sensitive ones, e.g., HCC827 and PC-9 (SF2 below 0.6) [55] (Figure 1A).

Next, EphA2 expression upon IR treatment was analyzed alongside DDR signaling components at 1 h post IR (Figure 1B). The IR-doses of 4 Gy and 8 Gy were chosen based on analyses of phosphorylation of DNA-PK_cs_ at S2056, ATM at S1981 and γH2AX in preparatory experiments (Figure S1). Phosphorylation of DNA-PK_cs_ at S2056, a site previously linked to its kinase activity after IR treatment [6,7,9] was evident in all cell lines (Figure 1B). The most prominent increase was observed in some of the most IR resistant cell lines (A549, U-1810), while the lowest activation was seen in the IR-sensitive H23 cells. Analyses of phosphorylation of the S1981 site in ATM [6,7,9] showed a clear increase in the most IR-resistant cell lines (H1299, A549 and U-1810), while similar to phosphorylation of DNA-PK_cs_, no clear change in ATM phosphorylation at S1981 was detected in H23 cells after IR (Figure 1B). At the time point when DDR signaling was active, EphA2 expression was evident in a majority of the NSCLC cell lines with minor differences seen prior to and post IR at this time point (Figure 1B). Thus, our results show that the expression of EphA2 correlates with IR refractoriness and there is some relationship between activation of the DDR signaling and expression of EphA2.

### 3.2. Silencing of EphA2 Expression Increases IR-Induced Apoptotic Signaling and Sensitizes NSCLC Cells to IR

**Figure 2:**
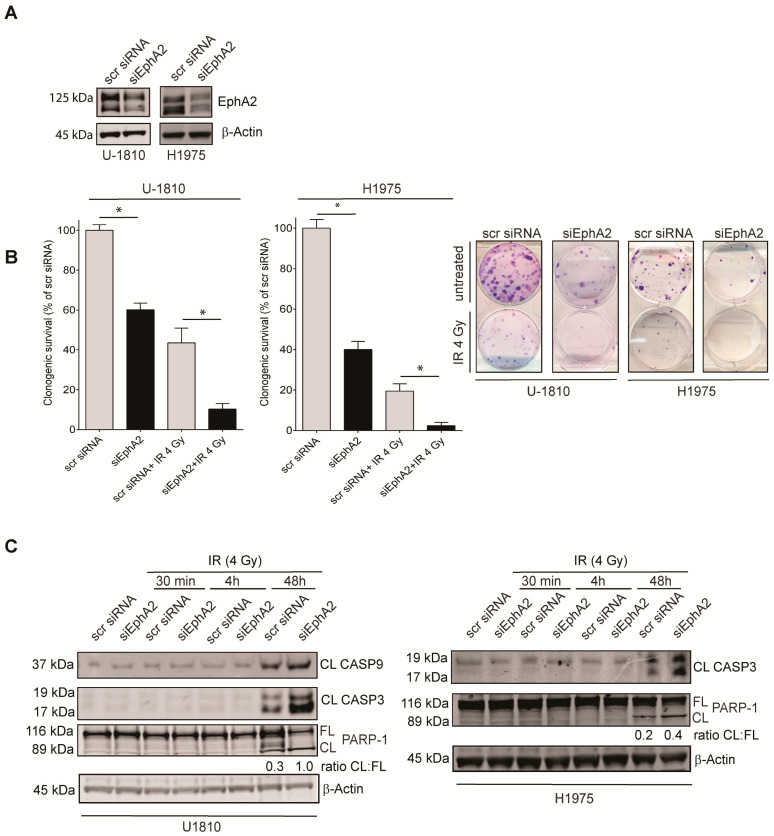
Silencing of EphA2 in non-small cell lung cancer (NSCLC) potentiates ionizing radiation (IR)-induced apoptotic cell death and reduces clonogenic survival. (**A**) EphA2 expression was silenced in U-1810 and H1975 cells using siRNA. β-Actin was used as a loading control. (**B**) U-1810 or H1975 cells were transfected with either scrambled (scr) siRNA or EphA2 siRNAs (siEphA2) for 24 h, irradiated (4 Gy) and thereafter seeded in 6-well plates for colony formation assay. The results are presented as percentages of colony survival in relation to non-irradiated scr siRNA transfected cells. Mean and SD bars are presented from one transfection of cells that were trypsinized, counted and seeded for colony formation in three independent dishes. Statistical significance was calculated by *t*-test, and *p* < 0.05 is indicated by *. Pictures of cell colonies transfected with indicated siRNAs with or without irradiation (4 Gy) are shown. (**C**) Cleavage of PARP-1, processing of caspase-3 and caspase-9 in samples from (**A**), which were irradiated (4 Gy) at 24 h post silencing. The cells were collected at 30 min, 4 h or 48 h post IR and analyzed for full length and cleaved PARP-1, cleaved caspase-3 and cleaved caspase-9 with β-Actin used as a loading control. Densitometry analysis was performed and the ratios of cleaved fragment of PARP-1 (CL) to its full length (FL) form are shown.

It has previously been shown that EphA2 influences NSCLC cell signaling and has an impact on their response or refractoriness to EGFR-TKI treatment as well as upon targeting tumor cell expressed VEGFR2 [25,26,27,28,30,31,32,33,34]. Given this and the results presented in Section 3.1, which showed that EphA2 expression was evident during IR-induced DDR signaling we next aimed to reveal if reducing EphA2 expression influenced sensitivity of NSCLC cells to IR. Thus, the expression of EphA2 was targeted with specific siRNA in NSCLC U-1810 and H1975 cells (Figure 2A).

To prove the significance of targeting EphA2 expression for cell survival, we analyzed the clonogenic capacity of U-1810 or H1975 cells with silenced EphA2 alone or in combination with IR (Figure 2B). In line with previous reports [27,28,32], silencing of EphA2 *per se* reduced colony formation capacity of both cell lines (Figure 2B), while when EphA2 knockdown was combined with IR, a further reduction in clonogenic survival was found, demonstrating an IR sensitizing effect of EphA2 ablation (Figure 2B). To assess if silencing of EphA2 influenced IR-induced activation of apoptosis, caspase-mediated cleavage of PARP-1 into a 90 kDa form was analyzed (Figure 2C). An approximate two to three-fold increase in the ratio of cleaved to total form of PARP-1 was observed in both cell lines when silencing of EphA2 was applied in combination with IR. Such increased PARP-1 cleavage in cells with silenced EphA2 expression was associated with increased caspase-3 processing. Moreover, in U-1810 cells activation of caspase-3 was also accompanied by an increase in the processed form of caspase-9, further suggesting the involvement of the mitochondrial apoptotic route in amplification of the apoptotic signaling (Figure 2C). Altogether, these data suggested that targeting EphA2 increased the cell death-inducing effect of IR in NSCLC cells.

### 3.3. EphA2 Localizes to Nucleus in NSCLC Cells Prior to and Post IR

**Figure 3:**
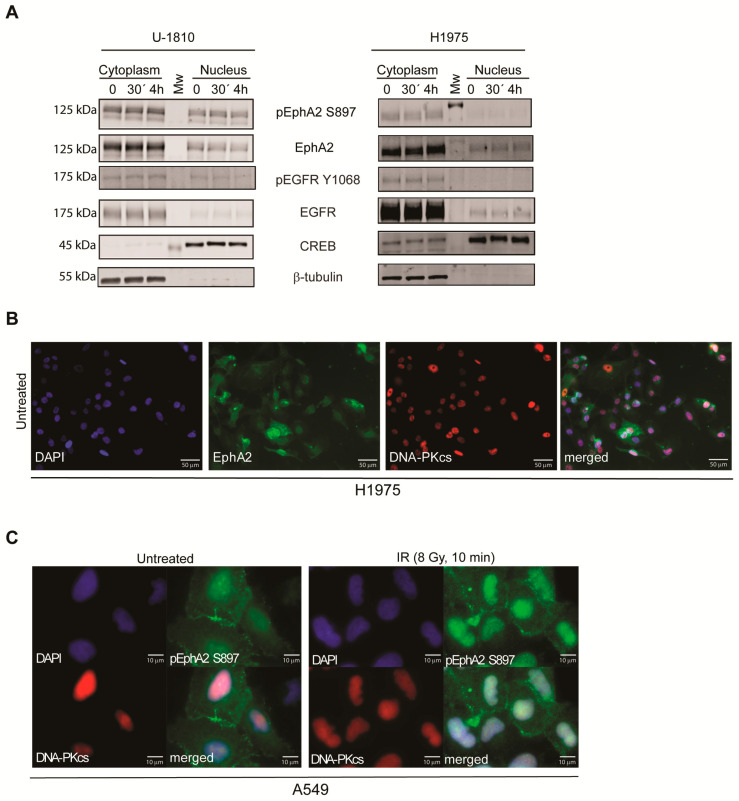
EphA2 localizes to nucleus prior to and post IR. (**A**) U-1810 or H1975 cells were irradiated with 8 Gy and harvested at 30 min or 4 h post IR. Cells were fractionated into cytosolic and nuclear fractions and analyzed by immunoblotting for expression of pEphA2 S897, total EphA2, pEGFR Y1068 and total EGFR. Expression of cAMP response element-binding protein CREB and β-tubulin was used to determine purity of nuclear and cytosolic fractions, respectively. (**B**) H1975 cells were stained with EphA2 and DNA-PK_cs_ antibodies. Cell nuclei were counterstained with DAPI. (**C**) A549 cells were irradiated (8 Gy) and at 10 min post IR fixed and analyzed for pEphA2 S897 or total DNA-PK_cs_. Cell nuclei were counterstained with DAPI.

The Eph family member EphA5 has earlier been reported to localize to the nucleus where it interacts with the DDR protein ATM [36]. Moreover, EphA2 was recently reported to have increased S897 phoshorylation after IR in NSCLC cells [52]. Given this report and our findings that showed that EphA2 expression correlated to IR sensitivity, influenced IR-induced cell death and clonogenic survival potential (Figure 1A and Figure 2B), we hypothesized that EphA2 may have some functions in DDR signaling. Therefore, EphA2 expression was analyzed in cytoplasmic and nuclear fractions of NSCLC cells prior to and post IR (Figure 3A) alongside the DDR proteins DNA-PK_cs_ and ATM (Figure S2). Both ATM and DNA-PK_cs_ kinases showed a strong phosphorylation within 30 min after IR (Figure S2). Interestingly, total EphA2 as well as pEphA2 S897 were both present in cytosolic and nuclear fractions of U-1810 cells (Figure 3A). In the time span analyzed, 30 min post IR no significant change in EphA2 or pEphA2 S897 protein distribution and/or expression levels was detected, while a lower expression of EphA2 in nuclear fraction (in relation to cytosolic fraction) was revealed in H1975 cells compared to U-1810 cells (Figure 3A).

EGFR has previously been reported to be expressed in cell nucleus [13,14,15] and here its expression in cytoplasmic and nuclear fractions was analyzed. As expected, a more prominent expression of EGFR was observed in the *EGFR* mutant H1975 cells, yet only trace amounts of EGFR were found in nucleus prior to or post IR (Figure 3A). To validate the localization of EphA2 in cell nucleus, cell immunostaining was performed. The results showed nuclear localization of EphA2 as seen by co-staining with DNA-PK_cs_ and DAPI (Figure 3B). Phosphorylated EphA2 S897 was, as expected, detected in the cell membrane but also evident in cytosolic and nuclear compartments in A549 cells both prior to and post IR (Figure 3C). Thus, our data revealed that EphA2 and its phosphorylated form EphA2 S897 partially localize to the cell nucleus prior to and post IR treatment.

### 3.4. EphA2 Interacts with DNA-PK_cs_ in Cell Nucleus

**Figure 4:**
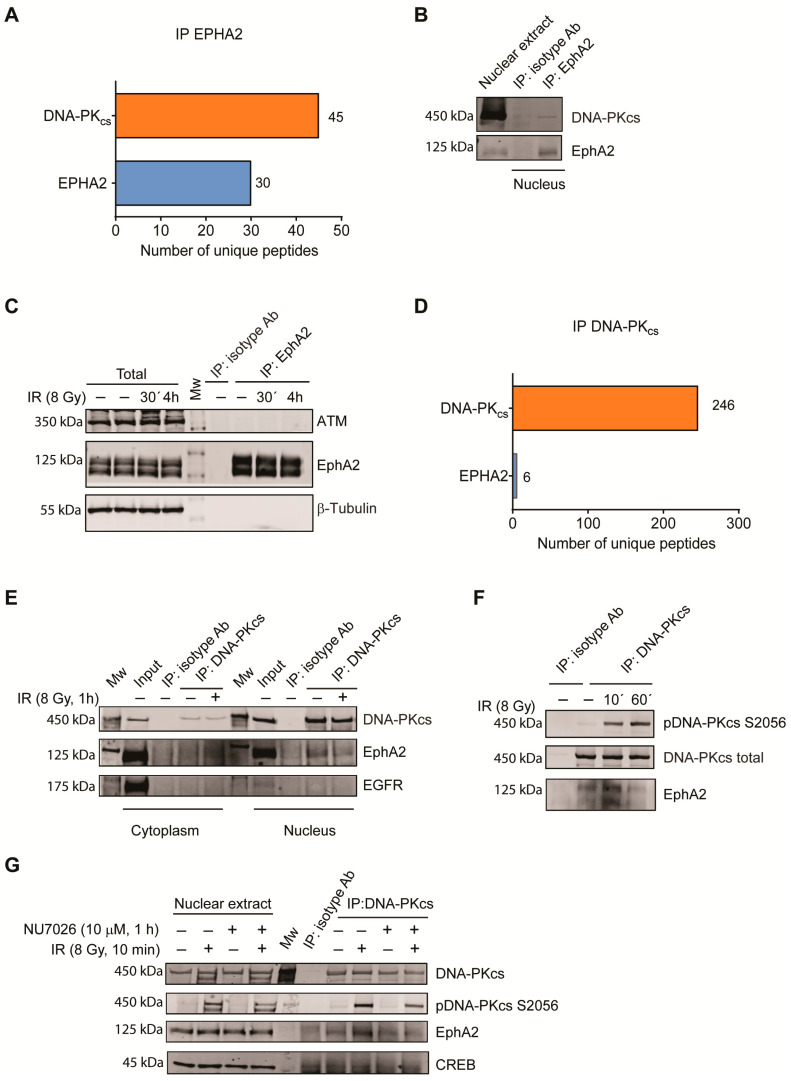
DNA-PK_cs_ interacts with EphA2 in cell nucleus. (**A**) EphA2 was immunoprecipitated (IP) from nuclear extracts of U-1810 cells. The number of EphA2 and DNA-PK_cs_ unique tryptic peptides identified by LC-MS/MS analysis is shown. The peptides identified for EphA2 or DNA-PK_cs_ are given in Data S1. (**B**) Nuclear extract was prepared from U-1810 cells and the extract volume was divided equally into two parts. IP was performed using either isotype control rabbit IgG or EphA2 antibody. Expression of DNA-PK_cs_ and EphA2 was analyzed by immunoblotting. (**C**) IP of EphA2 from U-1810 total cell lysates was performed using EphA2 antibody or rabbit isotype antibody control. Expression of ATM and EphA2 was analyzed by immunoblotting with β-Tubulin used as a loading control for total cell extracts. (**D**) DNA-PK_cs_ IPs from nuclear extracts of U-1810 cells. The number of EphA2 and DNA-PK_cs_ unique tryptic peptides identified by LC-MS/MS analysis is shown with peptide identity given in Data S1. (**E**) U-1810 cells were at 1 h post IR (8 Gy) fractionated into cytosolic and nuclear fractions. The fractions were subjected to IP with DNA-PK_cs_ antibody or mouse isotype antibody control. Protein concentration in each fraction was measured and equal amounts were subjected to IP. The IPs were blotted for total EphA2, DNA-PK_cs_ and EGFR. (**F**) A549 cells were treated with IR (8 Gy), and cells were collected at 10 and 60 min post IR. Nuclear extracts were prepared, and protein concentration in each extract was measured and adjusted to the same concentration. Equal volumes were used for IPs either with isotype mouse IgG or DNA-PK_cs_ Ab. The IPs were blotted for pDNA-PK_cs_ S2056, total DNA-PK_cs_ or total EphA2. (**G**) A549 cells were pre-treated for 1 h with NU7026 (10 µM) and thereafter exposed to IR (8 Gy). At 10 min post IR, nuclear extracts were made and subjected to IP with total DNA-PK_cs_. Total DNA-PK_cs_, pDNA-PK_cs_ S2056 or EphA2 were analyzed within the immunoconjugates or in the nuclear extracts. CREB served as a loading control.

As EphA2 was detected in the nucleus and showed correlation to the activation of DDR signaling, we next searched for potential EphA2 interactors that might be related to the DDR signaling and IR responsiveness. Thus, EphA2 was immunoprecipitated from nuclear extracts from U-1810 cells, and the immunoconjugates captured on beads were subjected to LC-MS/MS analyses to identify bound proteins. Results showed a EphA2 protein sequence coverage of 45%, demonstrating efficient IP. The LC-MS/MS analysis detected 45 DNA-PK_cs_ unique tryptic peptides (Data S1), thus identifying a novel interaction between EphA2 and DNA-PK_cs_ (Figure 4A). The association of DNA-PK_cs_ and EphA2 was further validated by immunoblotting (Figure 4B). In contrast to DNA-PK_cs_, the DDR kinase ATM that previously was reported to be an EphA5 interacting partner [36] was not detected in the immunoconjugates of DNA-PK_cs_, pointing towards a unique interaction of DNA-PK_cs_ with EphA2 (Figure 4C).

Next, we performed a reciprocal IP of the nuclear fraction using DNA-PK_cs_ antibody and similarly subjected the immunoconjugates to LC-MS/MS. In this analysis, an approximate 60% coverage of the DNA-PK_cs_ protein was achieved, and moreover, six EphA2 unique tryptic peptides were detected (Figure 4D, Data S1). To validate the MS data and further study, the EphA2 and DNA-PK_cs_ interaction, cell extracts from cytoplasmic and nuclear fractions of the U-1810 cells prior to and post IR were subjected to IP with DNA-PK_cs_ antibody in which EphA2 was analyzed by immunoblotting (Figure 4E). Moreover, a higher expression of EphA2 was evident in the nuclear fraction of U-1810 cells when compared to EGFR expression, which previously has been reported to interact with DNA-PK_cs_ [14,15]. To summarize, our data demonstrate a novel association between EphA2 and DNA-PK_cs_ in cell nucleus of NSCLC cells.

To verify that such interaction between DNA-PK_cs_ and EphA2 was not cell type specific, DNA-PK_cs_ was immunoprecipitated from A549 nuclear extracts prior to and post IR (Figure 4F). Similar to U-1810 cells, DNA-PK_cs_ was present in a complex with EphA2 prior to IR treatment in A549 cells (Figure 4F). The association between EphA2 and DNA-PK_cs_ was further increased shortly after IR exposure (10 min) but reduced at a later time point after IR (60 min). We next found that NU7026, an inhibitor of DNA-PK kinase activity, which also decreased DNA-PK_cs_ S2056 phosphorylation levels after IR, reduced the association between DNA-PK_cs_ and EphA2 after IR treatment, suggesting that impairing the kinase function of DNA-PK_cs_ may block the EphA2-interacting site on DNA-PK_cs_ (Figure 4G). To conclude, we report on a novel interaction of DNA-PKcs with EphA2 in cell nucleus.

### 3.5. Modulation of EphA2 Signaling Affects DNA-PK_cs_ Responses

**Figure 5:**
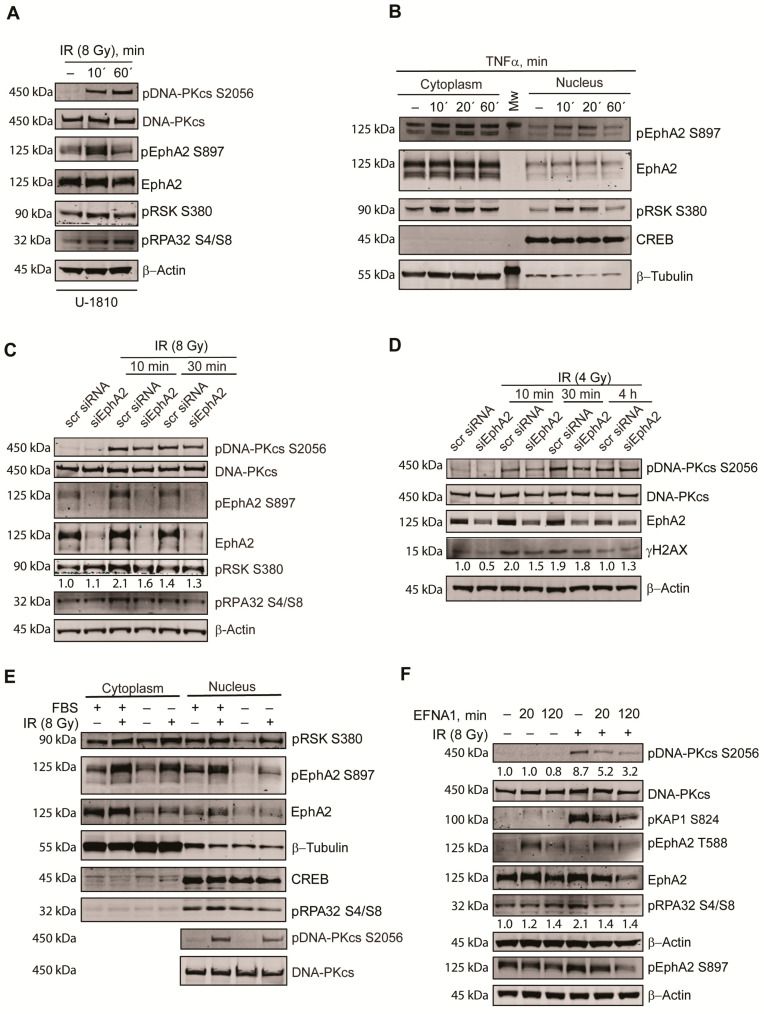
Modulation of EphA2 signaling affects DNA-PK responses. (**A**) U-1810 cells were irradiated (8 Gy) and 10- or 60 min post IR pEphA2 S897, pRSK S380, pRPA32 S4/S8 and pDNA-PK_cs_ S2056 were analyzed by immunoblotting together with the total forms of DNA-PK_cs_ and EphA2. β-Actin was used to control protein loading. (**B**) A549 cells were treated for indicated time with TNFα (20 ng/mL) and phosphorylation of EphA2 at S897, and RSK at S380 in cell fractions were analyzed by immunoblotting. β-Tubulin and CREB were used to control fraction loading. (**C**) Silencing of EphA2 was performed in U-1810 cells followed by IR treatment as indicated. The level of pDNA-PK_cs_ S2056, pRSK S380, pRPA32 S4/S8 and pEphA2 S897 are presented alongside total DNA-PK_cs_ and EphA2. The densitometric quantification of pRSK S380 is presented as a fold change relative to scr siRNA. Data shown are one out of two replicates. (**D**) EphA2 was silenced in U-1810 cells for 72 h followed by irradiation (4 Gy). Phosphorylation of DNA-PK_cs_ at S2056 and its total form as well as expression of EphA2 and H2AX phosphorylation at S139 (γ-H2AX) were detected by immunoblotting. β–Actin was used to control protein loading. The levels of γ-H2AX expression were quantified, and data are presented as a fold increase relative to scr siRNA. Data shown are one out of two replicates. (**E**) A549 cells were seeded and grown for 24 h in culture medium with or without fetal bovine serum (FBS) and then exposed to IR (8 Gy). Ten min after IR, cell fractionation was performed as described in Section 2.4. Expression of EphA2, its phosphorylation at S897, phosphorylation of RSK at S380, RPA32 protein at S4/S8 in cell fractions as well as the expression of DNA-PK_cs_ and its phosphorylation at S2056 in nuclear fraction were assessed by immunoblotting. β-Tubulin and CREB were used to control fraction loading. (**F**) A549 cells were treated with EFNA1 ligand for 20 or 120 min. In indicated samples cells were pretreated with EFNA1 ligand followed by IR (8 Gy) and samples were collected at 10 min post IR. Phosphorylation of EphA2 at T588 and S897 and its total form, DNA-PKcs and pDNA-PKcs S2056, pKAP1 S824 and pRPA32 S4/S8 expression were detected by immunoblotting. The densitometric quantification of pDNA-PK_cs_ S2056 and pRPA32 S4/S8 is presented as fold change relative to untreated cells after correction for loading differences among the samples using β–Actin. Data shown are one biological replicate with two different time points.

In order to reveal potential mechanisms of EphA2 activation that drive the interaction between DNA-PK_cs_ and EphA2 after IR, phosphorylation of EphA2 at S897 after IR exposure was studied alongside pDNA-PK_cs_ S2056 and phosphorylation of one of its substrates RPA32 at S4/S8 (Figure 5A).

A rapid (within 10 min) increase in S897 phosphorylation of EphA2 was observed, which almost returned to its basal level within 1 h post IR (Figure 5A). This result is also in accordance with data presented in Figure 3A, where no difference in pEphA2 S897 levels in U-1810 cells was evident between untreated and irradiated cells at 30 min post IR. The presented data is furthermore in line with earlier reports in NSCLC upon IR treatment [52] and illustrates that IR alongside EGFR- or VEGFR2 targeted therapy may influence EphA2 phosphorylation status on S897 [27,28,34].

The EphA2 S897 phosphorylation has earlier been reported to be controlled by RSK in tumor cells of different origins [30,57], including NSCLC [30,34,52]. In these earlier studies, RSK function was impaired by transfection of a kinase dead mutant or by pharmacological inhibitor BI-D1870, with a decreased phosphorylation of EphA2 at S897 being evident [30,34,52,57], indicating that it is the kinase function of RSK not the total protein expression that controls pEphA2 S897 levels. Next, we therefore studied if pRSK S380 also localized in nuclear fraction alongside pEphA2 S897, and similar to a previous report [30], EphA2 S897 phosphorylation was triggered by treating cells with TNFα (Figure 5B). Indeed, phosphorylation of RSK at S380 was kinetically associated with a concomitant increase in EphA2 S897 phosphorylation in the analyzed NSCLC cells, which was detected in both cytoplasmic and nuclear fractions. Moreover, as revealed by densitometry, an approximate two-fold increase in pRSK 380 was evident at 10 min after IR (8 Gy) (Figure 5C) and such increase in phosphorylation of RSK at S380 in response to IR treatment paralleled the increase in both total EphA2 and pEphA2 S897 expression (Figure 5C). Furthermore, we found that the IR-induced phosphorylation of RPA32 S4/S8 was reduced by about 50% at an early time point after IR, when EphA2 expression was silenced (Figure 5C). This alteration in phosphorylation of RPA32 was associated with changes in phosphorylation of DNA-PK_cs_ at S2056, which was also slightly reduced by silencing of EphA2 (Figure 5C).

Next, we analyzed whether a lower dose of IR (4 Gy) combined with silencing of EphA2 expression influenced DNA-PK_cs_-associated signaling (Figure 5D). Indeed, inhibition of EphA2 expression reduced the IR-induced increase in γH2AX levels at an early time point after IR as shown by densitometry with a concomitant reduction in DNA-PK_cs_ phosphorylation at S2056 (Figure 5D). Altogether our data suggest that in response to IR, phosphorylation of EphA2 at S897 stabilizes EphA2 protein expression by a yet unknown mechanism, leading to an increased interaction with DNA-PK_cs_, which influences the phosphorylation of some of DNA-PK_cs_ downstream substrates.

Following these results, we set out to analyze how alteration in EphA2 expression and activity affect DNA-PK signaling. Thus, phosphorylation of EphA2 was inhibited by culturing cells in FBS-free medium (Figure 5E). Deprivation of growth factors in culture medium reduced total EphA2 expression in both cytoplasmic and nuclear fractions, with a decrease in both RSK S380 and EphA2 S897 phosphorylation in nuclear extracts after IR. Moreover, such decreased expression of EphA2 was also associated with less phosphorylation of DNA-PK_cs_ at S2056 and RPA32 at S4/S8 in response to IR (Figure 5E). Multiple studies in tumor cells of different origin have shown that EFNA1 treatment leads to increased tyrosine phosphorylation of EphA2, e.g., at Y588 with a concomitant decrease in phosphorylation of EphA2 at S897 [37,40,41]. Given that pEphA2 S897 was in complex with DNA-PK_cs_, we next tested how alteration in EFNA1-mediated effects on EphA2 would influence its impact on DNA-PK_cs_ (Figure 5F). Indeed, a rapid EphA2 Y588 phosphorylation was evident in NSCLC cells treated with EFNA1 ligand, suggesting activation of the canonical signaling pathway. Moreover, the addition of EFNA1 to cells (for 120 min) blocked both baseline and IR-induced pEphA2 S897 levels, with a concomitant reduced stability of the total EphA2 protein (Figure 5F). In addition, EFNA1 treatment also imparted on the IR-induced phosphorylation of DNA-PK_cs_ at S2056, and quantification showed that the increase in phosphorylation of DNA-PK_cs_ at S2056 was reduced in the EFNA1 and IR combined treated samples at 20 min or 120 min (Figure 5F). This was also paralleled with a decreased phosphorylation of the DNA-PK_cs_ substrate pRPA32 S4/S8 in these samples. Similarly, the IR-induced increased level of pKAP1 S824, another substrate of DNA-PK_cs_, was blunted upon EFNA1 pretreatment (Figure 5F). Altogether, our data suggest that targeting EphA2 expression and activity affects DDR signaling and response to IR in part via DNA-PK_cs_.

## 4. Discussion

In the era of precision cancer medicine, where targeting of oncogenic drivers or using ICIs in NSCLC patients have reshaped the treatment landscape, there is still a large fraction of patients that will be given RT during the course of their disease. Given this it is important to understand the interplay between IR-induced DDR and RTK signaling, which may directly or indirectly affect cellular IR response. Further understanding of such interplay between DDR and RTK signaling may allow for novel RT sensitizing strategies.

**Figure 6:**
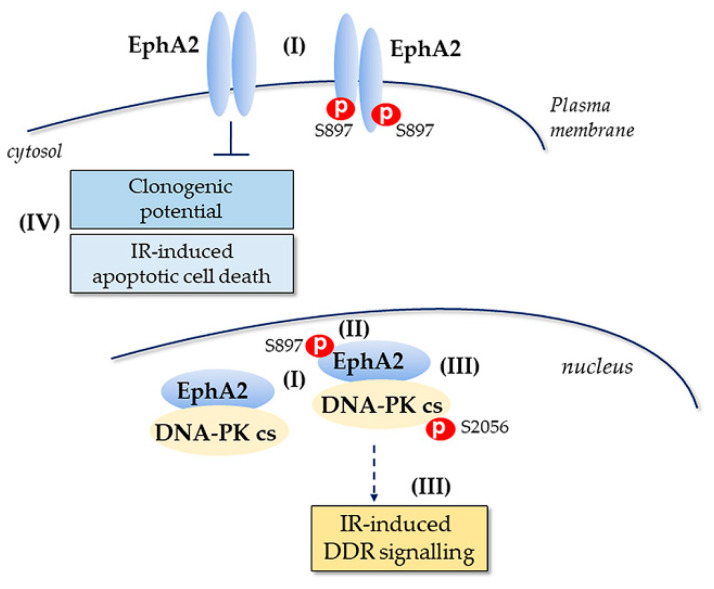
EphA2 interacts with DNA-PKcs in cell nucleus and influences IR-induced cellular response of NSCLC cells. Our data indicate that both total and EphA2 S897 are present in the cell nucleus prior to and post IR alongside localization in plasma membrane and cytosol (I). We observed a transient increase in EphA2 S897 early upon IR treatment and also found increased EphA2 total protein expression (II). We report on a novel interaction between EphA2 and DNA-PK_cs_ which may influence DDR signaling with the molecular details to be further studied (III) as well as on IR-induced clonogenic survival and cell death via the apoptotic route (IV). Thus, our findings indicate that in certain NSCLC cells, EphA2 alongside its effect on signaling events taking place at the plasma membrane/cytosol may also impact on IR-induced DDR signaling and highlight further a possible therapeutic potential of EphA2 for some NSCLC cases.

We and others have previously shown that EphA2 controls NSCLC cell survival, cell death, migration and response to EGFR-TKI or VEGFR2-targeting on tumor cells [25,26,27,28,29,30,31,32,34]. Other reports have demonstrated that both EphA and EphB receptors could influence tumor cell responses to IR [36,48,49,50] including EphA2 in NSCLC [52]. Therefore, we have in this study focused on the role of EphA2 in IR response of NSCLC cells, and in particular whether EphA2 could influence DDR signaling (Figure 6).

Our results showed a relationship between the EphA2 expression level and IR responsiveness in the NSCLC cell line panel analyzed. Moreover, we demonstrate that silencing of EphA2 expression reduced the clonogenic survival potential of NSCLC cells and sensitized for IR-induced apoptotic cell death. This IR-sensitization capacity of EphA2 ablation was evident in NSCLC cell lines of different histology and also in cells with mutant *EGFR*. These data on a role of EphA2 *per se* in the regulation of NSCLC cell survival are in line with earlier reports [26,27,28,31,32]. Our results are also in accordance with previous studies in *EGFR*-mutant NSCLC cells, where ablating EphA2 by genetic or pharmacological means could revert resistance to EGFR-TKIs [28,31].

We have earlier shown that deficient activation of mitochondrial apoptotic signaling is involved in IR resistance in NSCLC cells [58]. Moreover, we reported that silencing of EFNB3, a possible ligand of EphA2, sensitizes NSCLC cells to IR-induced apoptosis by engaging caspase signaling [47]. Given the observed increase in apoptotic signaling after EphA2 silencing and IR combined treatment in NSCLC, EphA2 may be put forward as a putative RT sensitizing target.

The interactome of EphA2 is complex, and focus thus far has been on proteins within the cytosolic compartment or at the plasma membrane, where two recent reports have identified PLCγ1 as well as VEGFR2 to form a complex with EphA2 influencing NSCLC cell survival and also metastatic potential [33,34]. Moreover, it is known that RTKs may directly associate with DDR signaling as illustrated for EGFR and DNA-PK_cs_, IGF-1R and NHEJ and more recently also for EphA5 and ATM [13,14,15,16,17,18,36]. Here, we for the first time show that EphA2, besides being expressed at the plasma membrane and in cytosol, localizes to the cell nucleus. By immunoprecipitation of EphA2 complexes and LC-MS/MS analysis, we revealed the association between EphA2 and DNA-PK_cs_, while no ATM was found in the complex with EphA2 in the analyzed NSCLC cells, which was previously shown for EphA5 [36]. Moreover, no other EphA/EphB peptides were identified in the DNA-PK_cs_ precipitates by LC-MS/MS analysis, suggesting a unique association of EphA2 with DNA-PK_cs_ over the other Eph family members, albeit this may be related to tumor cell type and different EphA/EphB expression levels. Our results are not in contradiction to earlier reports on the EphA2 interactors as indicated above but illustrate that, in addition to those interactions between EphA2 and membrane bound proteins, i.e., VEGFR2 [34], which influence NSCLC metastatic signaling, EphA2 may also impact on IR responses by influencing DDR. Thus, our data further illustrate that altering EphA2 signaling in NSCLC could have implications for multiple treatment regimens.

The formation of an EphA2 and DNA-PK_cs_ complex was evident prior to IR with a further enhancement shortly after IR exposure, suggesting that EphA2 could have an effect on some basal functions of DDR signaling with a more prominent effect seen early after IR, when an increased interaction to DNA-PK_cs_ is evident. Analyses in normal non-transformed cells and upon transformation into a malignant phenotype may further elucidate this. Our data show that accumulation of DNA-PK_cs_ and EphA2 complexes after IR exposure was linked to increased levels of EphA2 in the cell nucleus, which could be a result of EphA2 modifications. Further studies in which the protein stability of EphA2 is analyzed, e.g., by blocking protein translation, would be a path ahead.

As described above, phosphorylation of EphA2 at S897 is important for signaling status in different tumor cells including NSCLC [27,28,30,34,37,40,41]. In particular, it was shown that phosphorylation of EphA2 at S897 takes place after IR treatment of NSCLC cells [52]. Our data demonstrated that pEphA2 S897 was present in cell nucleus of NSCLC cells prior to IR with a rapid (10 min) increase upon IR exposure, which was then reduced within 30 min post IR. This effect was also in accordance with increased total EphA2 levels and a higher level of EphA2-DNA-PK_cs_ complexes. Thus, our data further point out the importance of EphA2 signaling in NSCLC cells and suggest a role of EphA2 also in cellular IR responses.

Multiple kinases are reported to control phosphorylation of EphA2 S897 in different tumor cell types at basal level and upon various treatments and among them RSK [30,34,52,57]. Thus RSK has been shown to phosphorylate EphA2 at S897 in response to TNFα treatment [30]. We here show that upon TNFα treatment of NSCLC cells both phosphorylated RSK and pEphA2 S897 partially localized to cell nuclei. Similar to TNFα treatment, RSK was rapidly phosphorylated in IR-exposed NSCLC cells—an effect which kinetically was also associated with phosphorylation of EphA2 at S897. We propose that such a rapid phosphorylation of EphA2 S897 regulates its stability in response to IR, leading to transient increase in EphA2 in cell nucleus. Yet further studies are required to understand the molecular mechanisms behind this. Our results also suggest that such an effect may facilitate the interaction between DNA-PK_cs_ and EphA2 with a subsequent potential impact on DNA-PK_cs_ downstream signaling. Moreover, the results with the DNA-PK_cs_ inhibitor suggest that DNA-PK_cs_ has to be in a certain conformation in order for the EphA2 to form a complex.

In our study we confirmed the existence of canonical and non-canonical activation of EphA2 that previously has been reported in different tumor types including NSCLC [20,21,22,23,27,28,30,31,32,34,37,38,39,40,41,42,43,44,45,46]. Thus, we show that treatment of NSCLC cells with recombinant EFNA1 increased phosphorylation of EphA2 at T588 with a concomitant reduction of both pEphA2 S897 and total EphA2 expression. Interestingly, pretreatment of NSCLC cells with EFNA1 ligand prior to IR also partially reduced DNA-PK_cs_ S2056 phosphorylation and accordingly phosphorylation of its downstream targets, i.e., KAP1 and RPA32. Thus, our results suggest that targeting Eph signaling with recombinant EFNA1 can affect DNA-PK_cs_-mediated IR responses by modulating EphA2 phosphorylation status. Moreover, in line with a protective function of EphA2 towards IR-inflicted DNA damages [52], we found that silencing of EphA2 expression in fact influenced IR-induced pRPA32 S4/S8 and pH2AX S139 status at early time points after IR exposure. As both these proteins are substrates of DNA-PK_cs_, results may suggest that EphA2 expression level possibly can boost DNA-PK_cs_ function after IR.

As described earlier, EphA2 has a clear impact on NSCLC signaling and influences targeted treatment responses including those elicited upon EGFR-TKI treatment or when targeting VEGFR2 expression on tumor cells [27,28,30,31,34,46]. Given these findings and the results presented in this study, it is relevant to think of therapeutic approaches towards EphA2 for RT sensitizing purposes. Thus far, no EphA2 interference strategies are used in the clinical setting of NSCLC, yet multiple ways to intrude with EphA2 signaling have been described for anti-tumor purposes, including antibodies, blocking peptides, kinase inhibitors and small molecules that act as antagonists or agonists towards EphA2 (reviewed in Baudet et al. [59] and Hughes and Virag [60]). These approaches may act by interfering with EFN ligand binding or by targeting the EphA2 kinase domain, thereby influencing the EphA2 oncogenic signaling. Thus, further studies on these approaches should be evaluated for RT sensitizing purposes, and the importance of EphA2 S897 phosphorylation site upon RT treatment of NSCLC patient tumors should be analyzed.

In summary, our data propose a novel function of EphA2, where its direct association with DNA-PK_cs_ influences cellular IR sensitivity and points towards a putative RT sensitizing target potential that could be used for pharmaceutical intervention.

## Data Availability

Presented data of this study will be available from the corresponding authors upon request.

## Supplementary Materials

The following are available online at https://www.mdpi.com/2072-6694/13/5/1010/s1, Figure S1: IR dose dependent activation of DDR signaling, Figure S2: Activation of DDR signaling in NSCLC cells, Data S1: The DNA-PKcs and EphA2 peptides identified by LC-MS/MS analyses.
