# Supplementary material for: EPHA2 Interacts with DNA-PKcs in Cell Nucleus and Controls Ionizing Radiation Responses in Non-Small Cell Lung Cancer Cells
Source: Cancers (Basel). 2021 Feb 28;13(5):1010. doi: 10.3390/cancers13051010 (PMC7957683; doi:10.3390/cancers13051010)
Supplement: Supplementary file 1 [file cancers-13-01010-s001.zip › Supplementary Fig S1-S2 Kaminskyy et al.pdf]

Supplementary Information to

EPHA2 Interacts with DNA-PK$_{cs}$ in Cell Nucleus and Controls Ionizing Radiation Responses in non-small cell lung cancer cells

Vitaliy O. Kaminskyy[1*], Petra Hååg[1], Metka Novak[1], Ákos Végvári[2], Vasiliki Arapi[1], Rolf Lewensohn[1,3], and Kristina Viktorsson[1*]

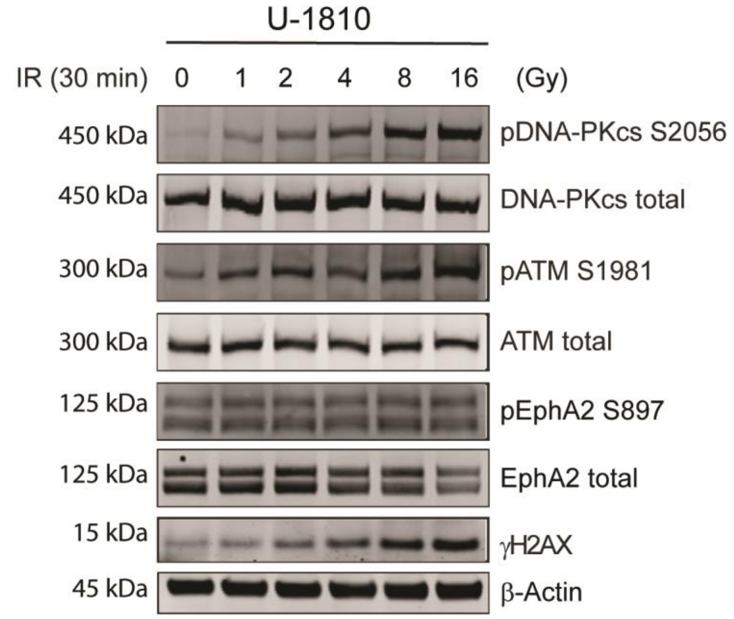

Supplementary Fig. S1. IR dose dependent activation of DDR signaling. U-1810 cells were irradiated with different doses and at 30 min profiled for the phosphorylation of DNA-PK$_{cs}$ S2056, ATM S1981, EphA2 S897, ◎H2AX or expression of total DNA-PK$_{cs}$, ATM or EphA2. To control for loading differences ß-actin was used.

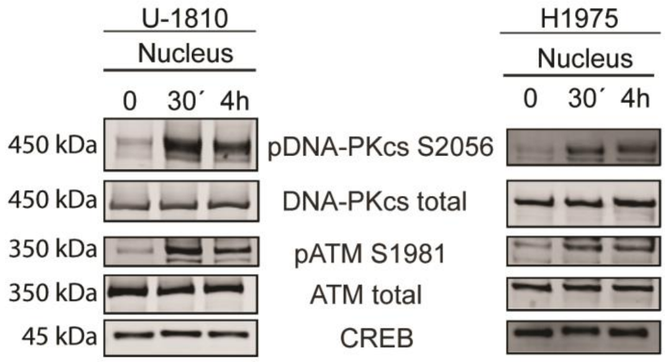

Supplementary Fig. S2. Activation of DDR signaling in NSCLC cells. Cells were fractionated into cytosolic and nuclear fractions and nuclear fractions analyzed by immunoblotting for expression of pDNA-PK_{cs}(S2056), pATM (S1981) and corresponding total proteins. Expression of CREB was used as a loading control.
